# Pseudo-Doctors

**Published:** 1847-09

**Authors:** 


*Pseudo-Doctors*.—The medical profession suffers not a little from the false assumption, or application of the title of doctorship. It is bad enough, in all conscience, to bear the disgrace inflicted occasionally by some of its legitimate members, and to be obliged to sustain the additional burthen of the misdeeds of those who choose to style themselves *Doctors*, or upon whom the generous and discriminating public chooses to bestow the title, is an imposition of which we may resonably complain. A short time since, among the newspaper items of the day which circulate over the whole country, it was announced that a certain Doctor somewhere in Michigan was arrested for the alledged crime of having poisoned his wife. Our esteemed contemporary, the Annalist, in commenting on the circumstance, gave utterance to some eloquent expressions of sorrow that the purity and nobleness of our profession should be sullied by such instances of criminal conduct in any of its members. Happening to be in conversation with one who resided in the neighborhood at the time, we learned that the individual referred to was not a regular physician, but a false pretender. An instance of a similar character has lately occurred in this city. A certain so called Dr. Allin has been committed to jail on the charge of a horrible attempt at crime upon the person of a young girl thirteen years of age. Notice of it will doubtless pass from paper to paper, and it will be generally inferred that the said Dr. Allin belongs to the Medical Fraternity of this city. This, however, is not the fact, his only known claim to the title of *Doctor* consisting in his having opened a shop for vending patent nostrums.

We think that it is but just to the medical profession, that Editors of papers in which such notices first make their appearance, take pains to state whether the individuals obnoxious to criminal accusation are regular members of the profession, or belong to the much more numerous class of *pseudo-doctors.*

